# The mechanism of human color vision and potential implanted devices for artificial color vision

**DOI:** 10.3389/fnins.2024.1408087

**Published:** 2024-06-19

**Authors:** Bingao Zhang, Rong Zhang, Jingjin Zhao, Jiarui Yang, Shengyong Xu

**Affiliations:** ^1^Key Laboratory for the Physics and Chemistry of Nanodevices, Institute of Physical Electronics, Department of Electronics, Peking University, Beijing, China; ^2^Beijing Key Laboratory of Restoration of Damaged Ocular Nerve, Department of Ophthalmology, Peking University Third Hospital, Beijing, China

**Keywords:** color vision, color perception, retina, visual cortex, artificial vision, restoration of sight

## Abstract

Vision plays a major role in perceiving external stimuli and information in our daily lives. The neural mechanism of color vision is complicated, involving the co-ordinated functions of a variety of cells, such as retinal cells and lateral geniculate nucleus cells, as well as multiple levels of the visual cortex. In this work, we reviewed the history of experimental and theoretical studies on this issue, from the fundamental functions of the individual cells of the visual system to the coding in the transmission of neural signals and sophisticated brain processes at different levels. We discuss various hypotheses, models, and theories related to the color vision mechanism and present some suggestions for developing novel implanted devices that may help restore color vision in visually impaired people or introduce artificial color vision to those who need it.

## 1 Introduction

Vision, the primary human sense, processes 80% of the information collected from the external environment. The intricate functions of the retina play a pivotal role in vision, integrating various information to facilitate comprehension of the external world. Unfortunately, 253 million people globally are deprived of this extraordinary sensory experience due to blindness, according to the World Blind Union.^1^ Various eye conditions, such as corneal injuries, retinitis pigmentosa, age-related macular degeneration (AMD), and diabetic retinopathy, frequently lead to vision impairment and even total blindness. These conditions present significant challenges for effective treatment using current medical technology, making vision restoration a distant goal.

Currently, the quest for vision restoration through artificial vision technology has become a hot research topic (Towle et al., 2021). A comprehensive understanding of the complex neural mechanisms regulating color vision is essential for developing advanced and efficient artificial vision systems. As technology evolves, the research and implementation of artificial visual prostheses are exceeding natural visual limitations, providing unprecedented opportunities for visual restoration for the visually impaired.

**Figure 1:**
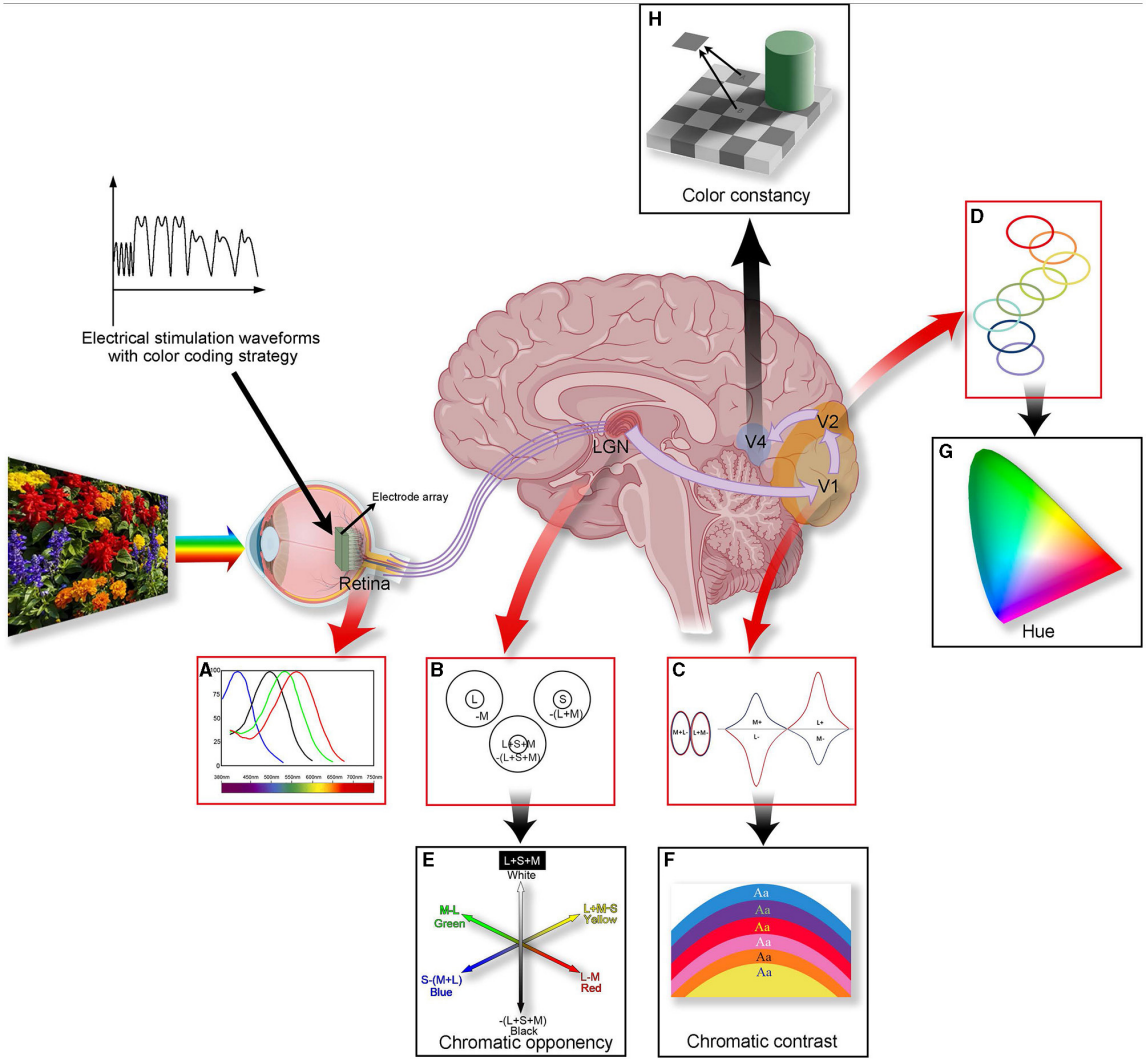
A summary of color processing in the color visual system. The figure illustrates the anatomy (the middle figure), physiology **(A–D)**, and perception **(E–H)** of color vision. Three types of visual cone cells (L, M, and S) capture information about different light frequencies based on their relative light absorption **(A)**. Cells within the LGN compare the cone signals **(B)**. Some cells' receptive fields are excited by L cone cells and are suppressed by M cone cells (forming “red-green” cells); others are excited by S cones and suppressed by (L + M) cones (forming “blue-yellow” cells), forming the basis for chromatic opponency **(E)**. In V1, specialized “double-opponent” cells compare cone ratios in specific visual spaces with those in adjacent areas **(C)**, probably constructed from LGN color cells, and they perform color contrast calculations **(F)**. The color signal then proceeds to V2, where specific hues are represented in color bands spanning the thin stripes of V2 **(D)**, potentially contributing to our perception of hue **(G)**. In V4, more cells will be associated with color constancy **(H)**. Processing color information involves multiple layers and is a complex process. Introducing coding strategies into artificial vision may lead to achieving controlled artificial color vision for the blind in the future.

Color vision holds a central role in human perception of external stimuli, offering precise object recognition and evoking profound emotional experiences (Valberg and Seim, 2008). However, the neural mechanism of color vision is complex, involving synergistic processes from the retina to the cerebral cortex (as shown in Figure 1). While physiological experiments can verify the process of vision generation, fully describing color vision perception remains challenging due to the complexity of neural mechanisms. A deeper understanding of this system requires not only meticulous experimental tools but also interdisciplinary research methods to reveal the basic principles of color perception (Pennartz et al., 2023).

It is widely accepted that color vision exploration starts with photoreceptors that perceive the primary colors, red, green, and blue, forming the foundational hues of the color world (Figure 1A). By using unique coding strategies, retinal ganglion cells (RGCs) transmit color information to the brain's higher structures. The retina and lateral geniculate nucleus (LGN) facilitate efficient color discrimination using complex transmission and opponent processes in the external environment (Figures 1B, E). The visual cortex in the brain is crucial for processing and integrating color information. Here, contrast (Figures 1C, F), hue (Figures 1D, G), and complex color vision information are deeply processed (such as color constancy, e.g., Figure 1H), forming our comprehensive perception of colors. The sophistication and efficiency of this system enable rapid and accurate human responses to rapidly changing environments.

For people suffering from visual impairment, traditional aids, such as canes, guide dogs, and braille, offer some assistance, but they do not fully replicate the rich informational experience offered by natural vision. Consequently, the development of visual aids, including wearable and implantable devices, especially artificial visual prostheses, has attracted significant interest. These advanced technological innovations aim to restore natural visual perception by emulating and replacing the impaired visual system, thereby providing an opportunity for individuals who have lost their sight to regain visual experiences (Guenther et al., 2012; Martino et al., 2013; Wang and Kuriyan, 2020; Lin et al., 2021).

Artificial visual prostheses are created by implanting electrodes or similar devices into the retina, optic nerve, LGN, or visual cortex. These devices capture external information and stimulate the retina with electrical signals to technically replicate natural vision functionality. Encouraging results have enabled some blind patients to regain limited visual perception (Hornig et al., 2017); however, contemporary artificial aids are significantly limited. Typically, they offer low-resolution, dichotic (black/white) vision, failing to provide realistic, natural visual experiences (Towle et al., 2021; Wu et al., 2023). Research in artificial visual technologies requires not only hardware and software innovations but also a deep understanding of color vision principles and mechanisms. This multidisciplinary challenge encompasses engineering, neuroscience, and computer science, aiming to accurately simulate the intricate process of color vision.

This paper aims to explore the neural mechanisms of color vision, ranging from basic visual cells to advanced brain information processing, to elucidate human color perception and understanding. In addition, We will analyze the contributions of artificial visual prostheses and their potential applications in color vision and suggest some new concepts for future artificial color vision technology. Current artificial vision devices restore some visual functions, but none specifically address controlled color vision restoration. This review aims to clarify the complex mechanism of color vision and establish a scientific foundation for developing artificial color vision technology. Ultimately, we hope to inspire researchers to create a controlled artificial color vision device. In this era of challenges and opportunities, we strive to advance artificial vision through our understanding of color vision mechanisms and innovative research in artificial color vision prostheses, thereby providing hope to those who seeking sight restoration.

## 2 Neural mechanisms of color vision

### 2.1 Trichromatic cones—The basis for the production of color vision

The study of color vision mechanisms has been vital in vision science for a long time. Artists have recognized for centuries that mixing three colors can yield a multitude of hues. Three primary colors act like orthogonal bases in linear space: none can be created by combining the other two, yet together, they can generate every color in the spectrum. Inspired by artists' color mixing, Young (1802) first proposed the trichromatic theory in the early nineteenth century. Maxwell (1857); Von Helmholtz (2013) later expanded upon this. This theory posits that human color perception relies on three primary color-receptive fibers: red, green, and blue. The theory's strength lies in explaining color mixing. It states that any color can be achieved by varying proportions of red, green, and blue. Color perception results from the simultaneous stimulation of three different light-sensitive fibers in particular ratios.

**Figure 2:**
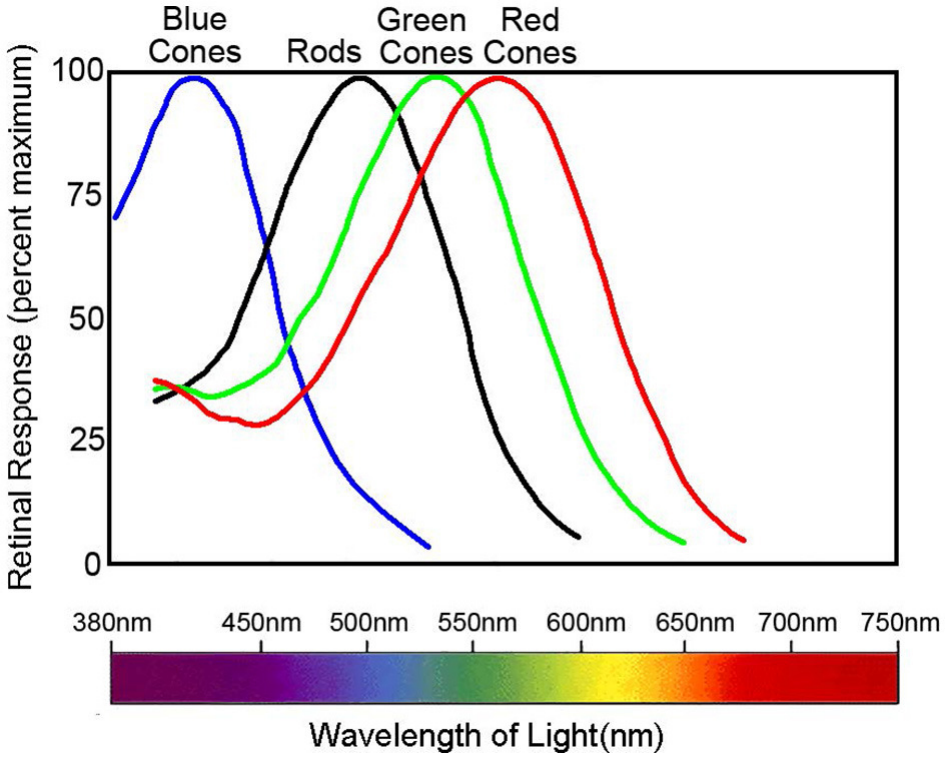
Normalized spectral sensitivity of retinal rod and cone cells. Data sourced from Nathans (1999). The peak absorption of the spectra by the three classes of cones, L, M, and S, is 560, 530, and 425 nm, respectively, whereas the peak absorption of the rods is around 500 nm. The figure illustrates the normalized spectral absorption peaks of the four cells. However, in fact, rods will only respond in very dark environments and depolarize in normal light intensity.

However, the trichromatic theory cannot completely explain all color phenomena. This includes negative afterimages and color blindness mechanisms. Current understanding shows that individuals with normal color vision possess three retina cone cells termed L, M, and S. These cones contain different photo-pigments sensitive to various wavelengths. Their absorption peaks correspond to 560, 530, and 425 nm, respectively (Figure 2) (Nathans, 1999). Organisms with a single class of photoreceptors cannot perceive color. This is because photoreceptor activation depends on both wavelength and stimulus intensity, even if there is a peak in the absorption spectrum.

Missing or anomalous cones can result in color blindness (Sharpe et al., 1999). The absence of a single type of cone can result in an inability to distinguish colors, which is easily understood. For instance, individuals with protanopia lack L-type cones, preventing them from perceiving red light, resulting in a tendency to see colors as shades of blue or gold. Individuals with protanomaly have all three types of cones present, but the L cones exhibit reduced sensitivity to red light. Red may appear as dark gray, and colors containing red may appear less bright. Colorblind glasses enhance color perception for individuals with mild forms of anomalous trichromacy. These glasses enhance color contrast, enabling individuals with color vision deficiencies to perceive color differences more distinctly. While there is currently no cure for most causes of color blindness, ongoing research is investigating gene therapy as a potential treatment for some severe cases (El Moussawi et al., 2021).

Indeed, L, M, and S cone cells are not evenly distributed across the visual field, as is desired for a screen display. Notably, in the retinas of primates and the majority of mammals, the density of S cones is significantly lower than that of L and M cones (Martin and Grünert, 1999). The distribution of S cones, constituting 5–10% of all cones, is well-defined (Curcio et al., 1991; Martin and Grünert, 1999; Hofer et al., 2005). The distribution of L and M cones, on the other hand, appears random. Additionally, individual differences exist in the ratio of L to M cones, which ranges from roughly 1:1 to 1:17 (Hofer et al., 2005). This was due to chromatic aberration. L cones detect both energy and wavelength contrast, while S cones are only dedicated to wavelength contrast. Chromatic aberration causes short-wavelength images to be out of focus when longer-wavelength images are in focus on the photoreceptor mosaic. This phenomenon enhances the dominance of the L cone system in energy contrast detection. Consequently, many mammals possess more L cones than S cones to enhance spatial resolution via the achromatic contrast detected by L cones (Gouras, 1995). Interestingly, the relative proportions of cones scarcely affect color vision. This implies that the brain utilizes adaptive and synthetic mechanisms to interpret color information from the signals provided by cones (Neitz et al., 2002; Jacobs et al., 2007).

For more technical details on the synaptic physiology and anatomy related to photoreceptors, one may read some excellent reviews in this field (Schmitz, 2009; Mercer and Thoreson, 2011; Regus-Leidig and Brandstätter, 2012).

### 2.2 Retinal ganglion cells—Visual encoding strategies

**Figure 3:**
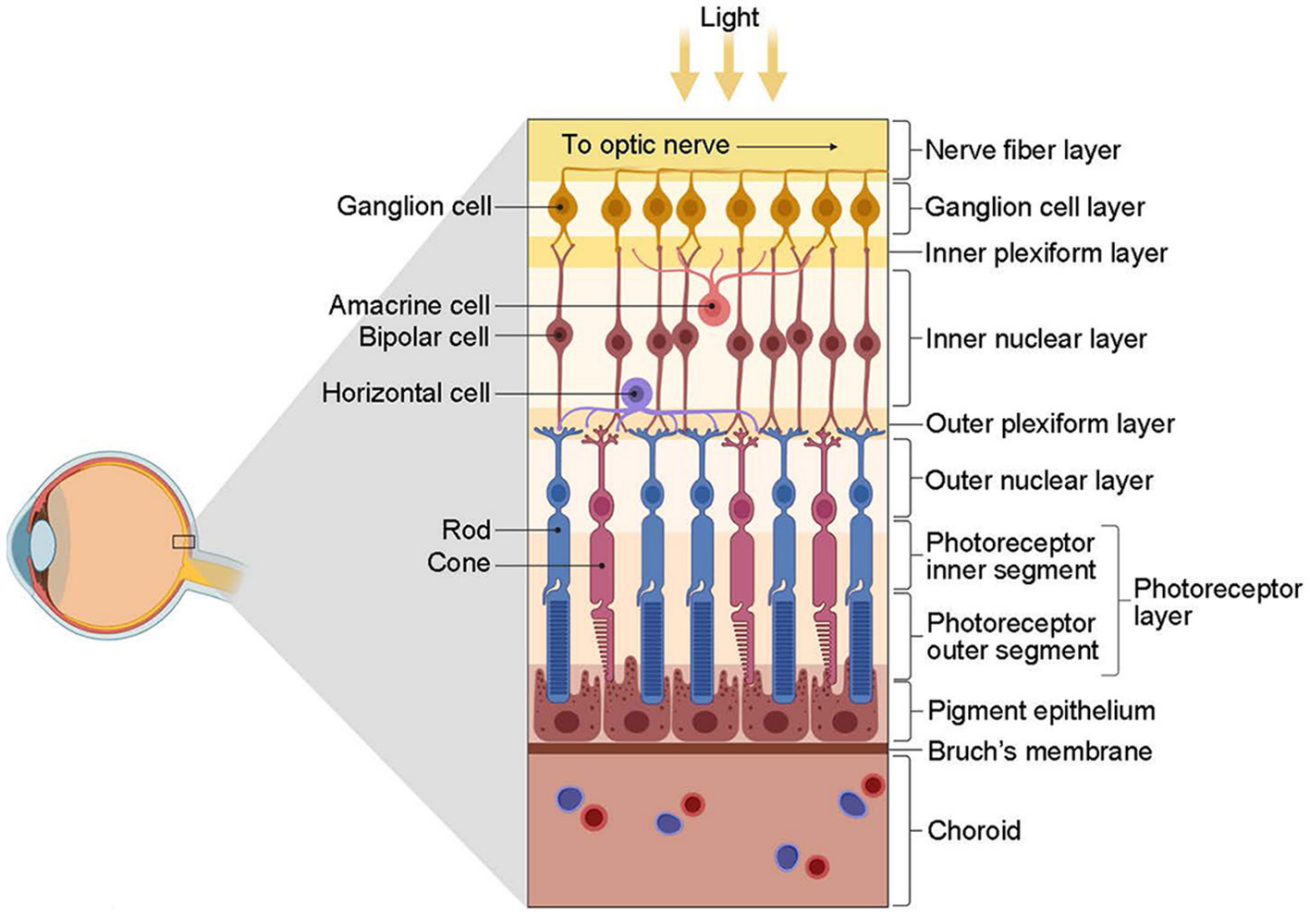
The structure of the retina (from Kim, 2020). The retina is primarily composed of three layers of neurons. The first contact with light is the ganglion cells, followed by the bipolar cells and, finally, the photoreceptors, including the cones and rods. However, photoreceptors are the first to respond to light. Interconnecting these three types of cells is two types of interneurons, namely, horizontal cells and amacrine cells. These cells have distinct roles and functions: photoreceptors receive light stimulation and convert it into electrical energy, eliciting nerve impulses; bipolar cells facilitate information transmission between photoreceptors and RGCs predominantly; horizontal cells and amacrine cells regulate the transmission of information from photoreceptors to bipolar cells and from bipolar cells to RGCs, respectively; RGCs are accountable for processing and integrating information, conveying electrical signals via nerve axons to the relay cells of the LGN, eventually reaching the visual cortex to engender vision. Created with BioRender.com.

The retina first encodes visual information integrally and then projects it to the LGN for further processing (Figure 3). In the outer plexiform layer, cones and rods use glutamate as a neurotransmitter that affects bipolar and horizontal cells. Bipolar neurons receive signals from photoreceptors and transmit them to RGCs. Horizontal cells and amacrine cells modulate the transmission of information from photoreceptors to bipolar cells and from bipolar cells to RGCs, respectively. RGCs process and integrate this information, then transmit electrical signals via neural axons to relay cells in the LGN. The encoding of information by RGCs represents the most crucial link in this process.

In 1926, Adrian and Zotterman (1926) discovered a positive correlation between the firing rate of sensory neurons in a frog and the pressure of touch stimuli. Subsequently, similar changes in firing rate were observed in the retina. For instance, when the stimulus brightness changed, the firing rate of tortoise RGCs also changed positively (Thiel et al., 2006). This correlation was similarly noted in rabbit RGCs (Risner et al., 2010). These findings suggest that frequency coding is a method by which the retina encodes visual information. However, later studies revealed that frequency coding alone is inadequate for processing visual information. It fails to explain the visual system's response to complex stimuli, such as primates' rapid recognition of faces and facial features. The cortex takes ~100 ms to respond to these stimuli after reception by temporal lobe neurons (Crouzet et al., 2010). However, visual information must pass through at least 10 synaptic transmissions from photoreceptors to these neurons. This implies that each processing stage cannot exceed 10 ms. Within these 10 ms, most neurons can only generate a maximum of one action potential (Thorpe et al., 2001). Consequently, neurons cannot rely on the time difference between two action potentials to determine the instantaneous firing rate, which rules out frequency coding alone.

**Figure 4:**
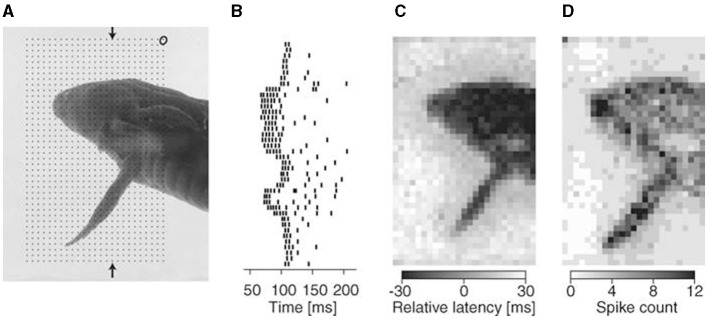
Visual stimulation is encoded by the response latency of retinal ganglion cells (Gollisch and Meister, 2008). **(A)** A photograph of a swimming salamander larva is projected onto the salamander retina. The ellipse in the upper right corner illustrates the receptive field of a salamander ganglion cell. During each stimulation trial, the image is slightly shifted based on the grid of dots. This allows for recording the responses of the depicted ganglion cell to all pixels in the photograph. **(B)** The firing activities of the depicted ganglion cell are illustrated, with each dot representing a spike at receptive field locations along the column, indicated by the arrows in **(A)**. **(C)** A visual image is reconstructed using a gray-scale plot based on the spike latency in each trial. **(D)** Corresponding gray-scale plot of the spike counts.

Besides frequency, RGCs encode time series features in their action potentials. A key aspect is the response delay of RGCs. Research indicates that stimulus intensity modulates the response delay of RGCs. Specifically, higher brightness or contrast results in a shorter response delay (Levick, 1973; Risner et al., 2010). Additionally, researchers discovered that the delay in the first action potential from RGCs encodes the spatial information in the stimulus image rapidly, as shown in Figure 4 (Gollisch and Meister, 2008). Considering this response delay, the recognition accuracy for various brightness and color stimuli significantly improved in experiments (Fernández et al., 2000; Greschner et al., 2006). Moreover, the duration of stimulation also affects neuronal response delay (Xiao et al., 2014). Beyond response delay, RGC activity includes other temporal features, such as the time difference between action potentials, which is crucial for encoding visual information (Rullen and Thorpe, 2001).

In addition to time-scale features, the synchronous response correlation of RGCs may encode color and other visual information. For instance, in frogs, the synchronous oscillatory activity of RGCs strongly correlates with visual processing in escape responses (Ishikane et al., 2005). This synchronous activity among visual neurons depends on specific features of visual stimuli. Although this activity is important for integrating sensory information, its exact importance and purpose have not been completely comprehended. Another example involves chickens' retinas, where single neurons show a positive correlation with light intensity (Chen et al., 2004). This correlation strengthens with red or green light but weakens with yellow or white light. This suggests that the collective activity of RGCs might play a role in processing and encoding color information. This specifically influences the processing of red and green light information. Researchers discovered that the correlation among bullfrog RGC populations can distinguish grid, horizontal raster, and vertical raster stimuli (Jing et al., 2010). This is notable because individual neuron firing patterns do not significantly vary among these three stimulus types. The receptive fields of adjacent RGCs are also adjacent. Thus, the correlated firing of neuron populations might encode more detailed spatial information (Pillow et al., 2008). The interaction within the higher levels of neuron populations results in group encoding complexity that surpasses the encoding capability of a single neuron.

RGCs employ various methods to encode visual information. This diversity equips the nervous system with effective tools to adapt to complex visual information. This is reflected in several key aspects: first of all, more efficient information transmission; despite being few in number, retinal RGCs deal with highly diverse visual information. Multiple coding methods enable these cells to transmit visual information efficiently, ensuring vital information reaches the posterior visual center effectively. Secondly, we consider adaptation to different stimulus conditions; various stimuli may necessitate different coding methods. RGCs can flexibly switch coding modes to adapt to diverse visual stimuli such as brightness, color, and movement, enabling them to more comprehensively encode visual scenes. Thirdly, this activity reflects biological information processing diversity; various coding methods offer diverse information-processing approaches. For instance, frequency coding, time characteristic coding, and correlation coding provide information from different perspectives during transmission, enhancing the nervous system's flexibility in processing visual information. Finally, using multiple encoding methods simultaneously can greatly enhance the accuracy of stimulus recognition. When considering factors such as firing rate, response delay, and group activity among neurons, retinal RGCs achieve more precise recognition of visual stimuli, leading to refined and comprehensive information processing. These flexible coding strategies guarantee the retina's accuracy and efficiency in transmitting visual information despite a limited number of nerve cells.

### 2.3 Retina and lateral geniculate nucleus—Color opponency

Following light excitation, rods and cones are responsible for modifying the release of glutamate vesicles at synaptic terminals. This changes the membrane potential of subsequent cells, including bipolar cells. In vertebrates, the opponency starts at the first synapse. Here, horizontal cells control the reciprocal inhibition of photoreceptors, enabling two-way information exchange. Opponency is a neural computational rule; it involves comparing a neuron's activity to a stimulus parameter, leading to inhibited neuronal activity. Hering (1878) originally proposed this model. He posited that color perception arises from opposite color mechanisms and that our visual system contains opposing color channels such as red/green, blue/yellow, and black/white. Hurvich and Jameson (1955, 1957) have made more improvements to the color opponency concept in the contemporary period, demonstrating that the perception of red can be counterbalanced by green and yellow. Color opponency serves as an effective mechanism for eliminating redundancies from overlapping the spectral sensitivities of various photoreceptor cells and reducing spectral redundancies in natural images (Buchsbaum and Gottschalk, 1983; Lee et al., 2002). This opposite feedback mechanism is prevalent throughout the visual system. The brain encodes and simplifies visual information by comparing visual signals through subtraction or proportional analysis.

**Figure 5:**
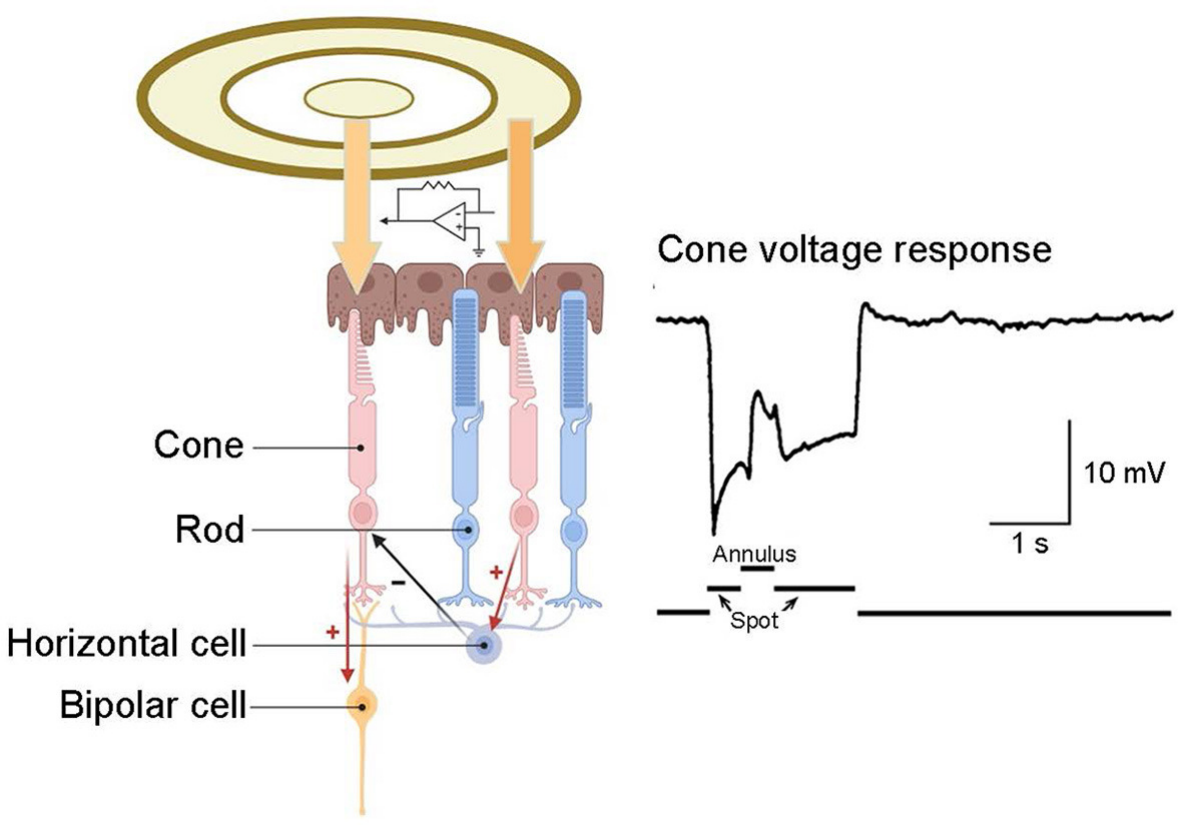
Inhibitory feedback from horizontal cells to cones. A recording from a turtle cone reveals that illuminating this cone with a small spot of light elicits a hyperpolarizing response. The subsequent application of an annulus to illuminate the surrounding receptive field induces hyperpolarization in the surrounding cones, leading to hyperpolarization in their postsynaptic horizontal cells. This alteration in inhibitory feedback from horizontal cells to the central cone elicits a depolarizing response in that cone. Cone response adapted from Burkhardt et al. (1988).

In the outer plexiform layer, glutamate released by cone pedicles depolarizes horizontal cells. The horizontal cells then provide negative feedback to the originating cone and surrounding cones (Figure 5) (Burkhardt et al., 1988; Twig et al., 2003; Thoreson et al., 2008). This interaction among cone cells and horizontal cells leads to color opponency at the visual system's first synapse. As a single photoreceptor cannot differentiate wavelength shifts from intensity changes, the opponent process occurs between at least two types of photoreceptors with different spectral sensitivities (Krauskopf et al., 1982). Horizontal cells typically have a wide receptive field, attributed to their large dendrites and strong intercellular coupling. Consequently, the horizontal cells' negative feedback to cones forms a broad opponent receptive field. This field surrounds a small, excited central region in the cone while inhibiting the surrounding region. This integrated, central-peripheral-receptive field information is relayed to bipolar cells and, ultimately, to RGCs. As previously mentioned, this mechanism operates by subtracting the average light level, as detected by horizontal cells across a broad area, from the light response of a central cone. This process efficiently removes spatially redundant brightness and color information, enhancing signal transmission efficiency (Kamermans et al., 2001; Jackman et al., 2011; Chapot et al., 2017).

The parvocellular pathway is primarily composed of midget RGCs. Functional evaluations reveal that their sensitivity to luminance contrast is lower compared to parasol RGCs, and most exhibit distinct chromatic opponency (Wiesel and Hubel, 1966). Typically, midget cells display red-green opponency, while parasol RGCs lack chromatic sensitivity, and bi-stratified ganglion cells link with S cone ON and L-M cone OFF pathways. Recent research suggests that certain OFF midget cells receive signals from short wavelength (blue)-sensitive cones (Tsukamoto and Omi, 2015; Wool et al., 2019). Electron microscopy reconstructions propose that a small fraction of midget ganglion cells may possess blue OFF and yellow ON receptive fields. Besides color discrimination, midget RGCs contribute to pattern recognition, texture discrimination, and stereoscopic depth perception (Schiller, 2010).

These complex signals undergo initial processing by bipolar cells, and this is then followed by further processing by various RGCs (Sernagor et al., 2001). Most RGCs transmit signals to the LGN via axons, and LGN cells relay these signals to the visual cortex through their axons. Under most neurophysiological experimental conditions, the conversion of visual signals between RGCs and LGN neurons is minimal and is typically considered a linear projection (Usrey et al., 1999). For instance, parasol RGCs project to magnocellular (M cells) neurons, while midget RGCs connect to small parvocellular (P cells) neurons. Additionally, small, bi-stratified RGCs and those with less defined characteristics, and these project to koniocellular (K cells) neurons (Hendry and Reid, 2000; Hashemi-Nezhad et al., 2008; Roy et al., 2009).

The color sensitivity of the three types of cells in the LGN varies based on cone opponency (De Valois et al., 2000). When a specific type of cone cell is activated, it triggers cone opponent cells to discharge at a frequency above a certain threshold. Conversely, the activation of another type of cone cell results in a reduced discharge frequency. Most neurons in the M cell layer are responsive to changes in luminance. Luminance is a combination of L and M cone signals and lacks color selectivity. Neurons in the P cell layer primarily focus on the difference between L and M cone signals (“L-M”), whereas neurons in the K cell layer respond to differences between S cone signals and the sum of L and M cone signals [“S-(L+M)”] (Hendry and Yoshioka, 1994). These three types of LGN neurons exhibit sensitivity to color changes in directions that closely align with the psychophysically defined “cardinal directions” of red-green, blue-yellow, and black-white in color space (MacLeod and Boynton, 1979; Krauskopf et al., 1982; Derrington et al., 1984). The combined activation and suppression of these opposing channels shape our perception of different colors. Cone opponent cells perform calculations that are essential for distinguishing between wavelength and intensity, serving as fundamental components in color vision (Devalois and Devalois, 1993).

Subsequently, each LGN projects to a specific region in the primary visual cortex (V1). Currently, the role of the LGN in processing color signals remains unclear. Our focus here is on cone opponent cells in both the retina and the LGN. When a specific type of cone cell is activated, it triggers cone opponent cells to discharge at a frequency above a certain threshold. Conversely, activation of a different type of cone cell results in a reduced discharge frequency. Various types of cone opponent cells exist, including red-green (L-M) cells, which compare L-type and M-type cone activations and blue-yellow (S-(L+M)) cells by comparing S-type cone activation with a combination of L and M cones (Dacey and Lee, 1994; Field et al., 2007). The combined activation and suppression of these opposing channels shape our perception of different colors. Cells activated by L cones are often termed “red-on” cells, while those activated by M cones are called “green-on” cells, and so on. Cone opponent cells perform calculations that are essential for distinguishing between wavelength and intensity, serving as fundamental components in color vision (Devalois and Devalois, 1993).

### 2.4 Visual cortex—Processing contrast, hue, and higher-order color information

#### 2.4.1 V1: chromatic contrast

After forming synapses in the LGN, visual information proceeds ventrally to the V1 in the occipital lobe. Within V1, diverse neurons specialize in processing various aspects of visual information. For instance, some neurons are responsive to color, orientation, motion direction, edge, and spatial frequency. Electrophysiological recordings often show local potential responses indicating red-green sensitivity in both the supra- and subcortical regions of V1. In layers II and III of V1, up to 64% of the cells are color-selective (Friedman et al., 2003). Studies employing magnetic resonance imaging (MRI) to analyze the human V1 have shown strong responses to red-green and yellow-blue tests (Engel et al., 1997). All these findings further support the role of the V1 region in color encoding.

Historically, it was believed that color, motion, and shape were processed in parallel by distinct modules within the visual cortex. This concept of modularity originated with Hering and was further developed by Hurvich and Jameson (1957). Krauskopf and colleagues later adopted this modular approach in their study of the fundamental directions of color space (Krauskopf et al., 1982). Unlike the spatial band-pass contrast sensitivity of luminance patterns, the red-green isoluminance pattern's contrast sensitivity is low-pass. This supports the modular view (Mullen, 1985). Zeki (1973, 1978a,b) later emphasized this modular view in his functional studies of the visual cortex's striations. In Zeki's studies, he highlighted that different striate areas in the macaque cortex specialize in various visual features. For example, cortex V5 predominantly comprises neurons responsible for direction selectivity, while region V4 is rich in neurons responsive to color.

**Figure 6:**
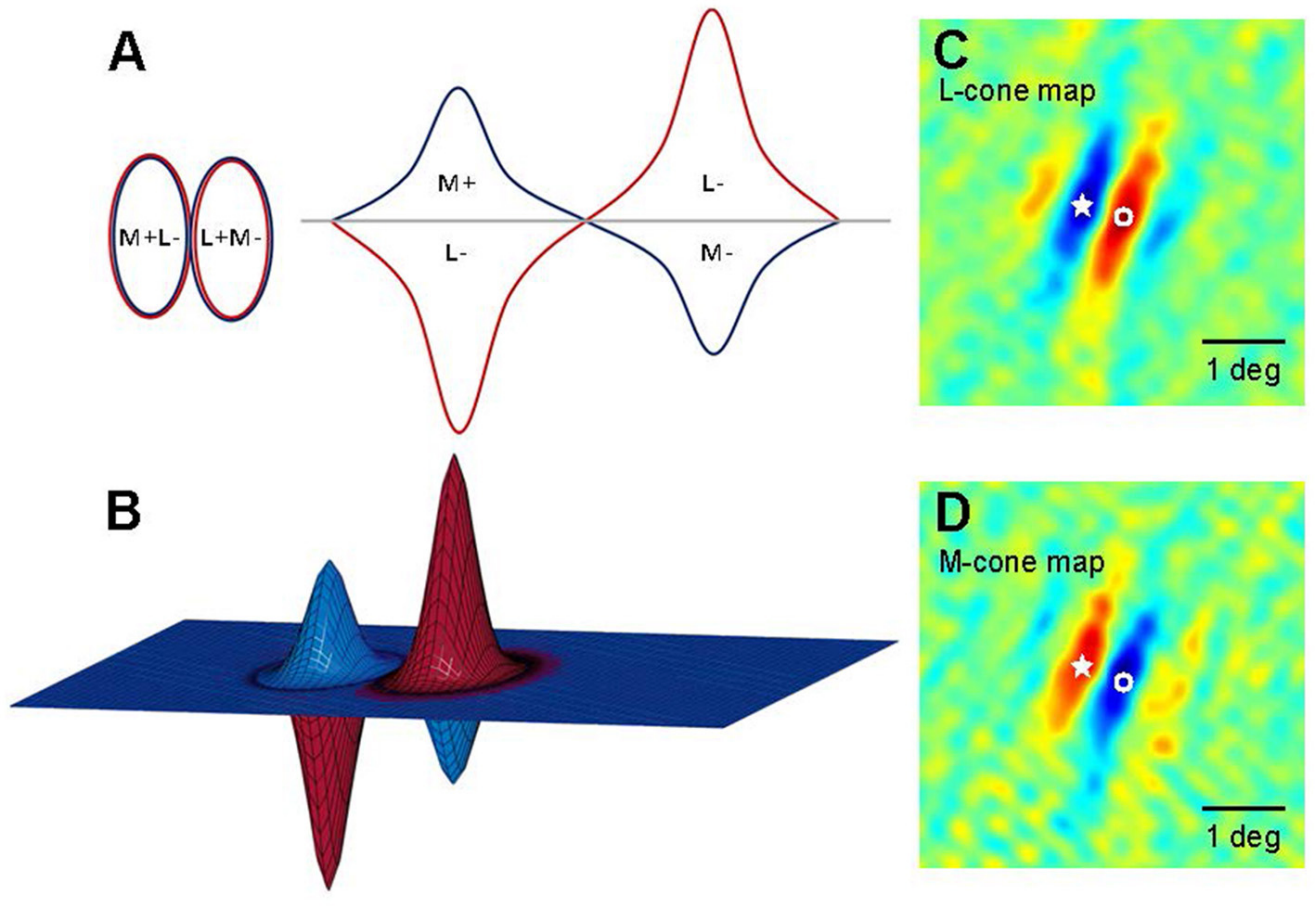
Models of double-opponent V1 neurons. **(A)** A schematic representation of receptive fields is depicted for a 2D plane of L-M opponent cells, exhibiting side-by-side spatial antagonistic regions and opponent cone weights. Weights above the horizontal plane indicate “ON” states, wherein an increase in light elicits an increase in response; conversely, weights below the horizontal plane signify “OFF” states, where a decrease in light leads to a decrease in response. The left panel illustrates the organization of the 2D receptive field, while the right panel presents a hypothetical spatial sensitivity profile. **(B)** A 3D schematic of the aforementioned receptive field model. **(C, D)** Two-dimensional maps (derived from subspace reverse correlation) depict the sensitivity of this cell to L (A) and M (B) cone isolation patterns (Johnson et al., 2008). Pseudo-color mapping indicates excitation to increases in red and excitation to decreases in blue. Fixation points within the visual field are denoted by stars and open circles to facilitate comparisons between L cone and M cone maps. At the star locations, L cone maps exhibit decreasing excitatory responses, whereas M cone maps show increasing excitatory responses; conversely, at locations labeled with open circles, the pattern is reversed.

Livingstone and Hubel's (1984, 1987, 1988) work on areas V1 and V2 also supports modularity, linking it to the parallel processing of color and shape in the LGN. In addition, Livingstone and Huble identified double-opponent cells in layers II and III of region V1, which is crucial for color perception. These cells are termed “double opponent” due to their opposite integration of antagonistic wavelength information from different cones and spatial information from various receptive fields. These cells have concentric receptive fields, activated or inhibited by central red light and surrounding green light, as shown in Figure 6. They respond more intensely to color boundaries and patterns than to extensive color areas (Livingstone and Hubel, 1984; Thorell et al., 1984; Johnson et al., 2001, 2008). Contrast is a key aspect of color perception, which likely relies on the signals from double-opponent cells. Psychophysical masking and adaptation experiments conducted in isoluminance mode indicate that double-opponent cells play a crucial role in the spatial frequency tuning of color channels (Bradley et al., 1988; Switkes et al., 1988; Losada and Mullen, 1994; Vimal, 1997; Beaudot and Mullen, 2005).

The modular view suggests that while color vision functions may be less adept at processing shape, shape vision functions predominantly depend on luminance signals. However, psychophysical studies challenge this modular model by demonstrating similar thresholds in orientation discrimination for both color and luminance stimuli (Webster et al., 1990; Beaudot and Mullen, 2005). Similarly, the contour integration performance from local elements, whether of color or brightness, is comparable (McIlhagga and Mullen, 1996; Rentzeperis and Kiper, 2010). Additionally, recent experiments assessing changes in the orientation and color of two stripes have been inconclusive regarding the separability of color and orientation (Bimler et al., 2013). These lines of evidence suggest a potential interaction between color and spatial orientation mechanisms in the early stages of visual processing.

#### 2.4.2 V2: hue

As early as 1984, Hubel discovered color-sensitive cytochrome oxidase (CO) blobs in area V1. Initially identified due to their affinity for cytochrome oxidase staining, the cells in these spots encode color information and project to the CO region of V2. This suggests a high concentration of color-selective neurons in the V2 cortical layers. Researchers estimate that over half of V2 neurons are color-sensitive, with a minority being selective for size and direction. There is no significant variance in the distribution of color-selective cells across different CO areas, nor a negative correlation between color selectivity and other attributes.

In V2, color-selective cells tend to be more concentrated in the thin stripes (Lu and Roe, 2008). Functional magnetic resonance imaging (fMRI) has also demonstrated clearer functional differentiation within these thin stripes (Conway et al., 2007). Individual neurons in V2 carry considerably less color information compared to those in V1. Despite this, there is no significant difference in color coding between V1 and V2 (Kiper et al., 1997; Solomon and Lennie, 2005).

**Figure 7:**
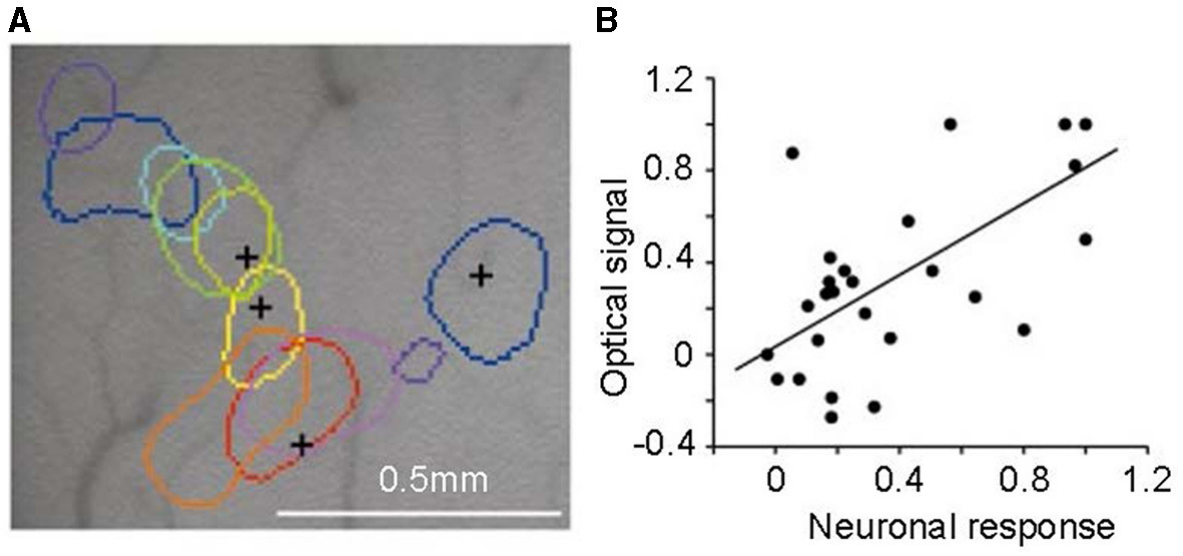
Color-specific band in the second cortical visual area (V2). **(A)** Regions corresponding to peak activity in response to different colored stimuli, each tested independently, are delineated on the surface image of the brain. **(B)** The correlation between neuronal responses recorded by multi-unit electrodes and optical signals in V2 color bands reveals a significant correlation between the two signal types. Adapted from Xiao et al. (2003).

By using optical imaging techniques, Xiao et al. (2003) discovered an intriguing phenomenon. They employed a sensitive camera to detect changes in tissue optical density indicative of activity in the V2 area. Responses to colors, including red, orange, yellow, greenish-yellow, green, cyan, blue, and purple, were measured. These colors were presented as isoluminance color-and-gray gratings. Altering the order of these gratings showed that the peak activity location in the V2 cortex also shifted sequentially for each color (shown in Figure 7). The area most responsive to red was adjacent to that for orange, followed by yellow, green, etc., creating a trend in peak activity locations corresponding to spectral frequency. This suggests a potential role of V2 in hue processing, though a definitive conclusion is yet to be drawn.

These findings lead to the question, What is the true meaning of the neural representation of color? Currently, researchers focus on describing the patterns of brain activity in vision. One benefit is the potential development of artificial vision and brain stimulation devices to restore color vision in individuals with impaired visual systems.

#### 2.4.3 V4: color constancy

Color constancy is a key feature of the V4 region. Color constancy means that although the wavelength composition of light reflected by an object changes under different lighting conditions, the perception is that the object maintains a constant color.

For instance, an apple remains red both in light and in shadow. Color and brightness constancy is also found in V1 and V2 (Conway, 2001; MacEvoy and Paradiso, 2001; Shapley and Hawken, 2002), and the effects are especially prominent in V4 (Zeki, 1983). Studies have shown that background lighting alters the tuning of V4 neurons (Kusunoki et al., 2006). The cells adjust their color-tuning function in response to changes in the chromaticity of the illuminant. This adjustment corresponds with psychophysical perception (Schein and Desimone, 1990). Consistent with these findings, damage to V4 affects color constancy perception, although color discrimination remains intact (Heywood et al., 1992; Vaina, 1994). A recent fMRI study revealed a gradient in the visual hierarchy, from V1 to V4α, progressively encoding surface color rather than illumination (Bannert and Bartels, 2018). This finding offers a principled characterization of color constancy mechanisms across the visual hierarchy and shows complementary roles in the early and late processing stages.

## 3 Artificial vision

Exploring color vision mechanisms offers profound insights. Integrating these into artificial color vision device research is expected to drive scientific and technological breakthroughs in simulating human visual perception. With an understanding of the principles and mechanisms of color vision, the next step is exploring their simulation and optimization in artificial vision.

### 3.1 Implantable visual-assistive devices

Artificial vision represents a technology crafted to partially re-instate visual functionality in individuals who are blind or possess severely impaired vision. Artificial vision research started in 1929 with German neurosurgeon Otfrid Forester, who first electrically stimulated an exposed occipital lobe in a patient (Foerster, 1929). In the 1980s, William Dobelle achieved some success with the design of the first cortical implant (Hornig et al., 2017). This development laid the physiological foundation for creating visual prostheses to restore vision.

In the past three decades, artificial vision devices that electrically stimulate surviving visual neurons have become a promising treatment for restoring vision in the blind. Based on their location of implantation, artificial visual devices are categorized into retinal prostheses, optic nerve prostheses, LGN prostheses, and visual cortex prostheses (VCPs). Typically, images of the surrounding environment are captured utilizing specialized eyeglasses or camera devices. Subsequently, these images undergo conversion into electronic signals, which are then conveyed either directly to the retina or the visual center through electrical stimulation or to the retinal sensory nerves via micro-photodiode arrays embedded within the eye. Eventually, the electrical stimulation signals are transmitted to the visual cortex of the brain, eliciting visual perception.

Most visual prostheses are primarily designed to address retinitis pigmentosa (RP) and age-related macular degeneration, the two leading causes of blindness. However, as research progresses, visual prostheses may also become a treatment option for other eye diseases, such as glaucoma and eye trauma. Generally, if the nerve pathways behind the RGCs remain intact, patients can use all types of artificial visual devices. However, damage to the optic nerve disrupts the transmission of visual signals to the visual cortex, rendering retinal and optic nerve prostheses unsuitable. In such cases, a VCP is preferable. Retinal implants and visual cortex implants are the preferred sites for visual prostheses due to their location at both extremes of the visual pathway, making them more surgically accessible compared to deeper brain structures, such as the optic nerve and LGN.

Optic nerve implants have demonstrated the ability to induce phosphene by longitudinally implanting electrodes into the optic nerve (Veraart et al., 1998). However, their resolution falls short compared to that of retinal prostheses and VCP. Furthermore, the positioning of the implant on the optic nerve at the rear of the eye introduces unpredictability in the localization of phosphene (Chai et al., 2008). In contrast to implants that do not rely on an intact optic nerve and retina, VCPs are directly placed within the early optic cortex. Despite the expansive area of the optic cortex resulting in a relatively high resolution of the stimulating electrode, predicting the mapping of phosphene within the visual cortex remains equally challenging (Niketeghad and Pouratian, 2019). Electrical stimulation in the visual cortex can also predispose to epilepsy.

Based on the location of implantation, retinal implants are classified into three types: epi-retinal, subretinal, and supra-choroidal. The epi-retinal implant, which is placed in the front of the retina, has its electrical stimulator in contact with the ganglion cell layer, directly stimulating these cells to elicit a cortical response. The subretinal implant, positioned beneath the retina, features an electrical stimulator that contacts the photosensitive layer. The supra-choroidal implant is situated between the sclera and the choroid. Both subretinal and supra-choroidal implants aim to replace degenerated photoreceptors and stimulate the remaining bipolar cells in the retina (Stingl et al., 2017).

Technologically speaking, these devices transmit to the patient's retina by converting light signals into electrical signals. Several devices have been implanted in human patients, including Second Sight's Epiretinal Argus II (da Cruz et al., 2013), EpiRet's EpiRet III (Roessler et al., 2009), the Alpha IMS/AMS of Retina Implant AG (Stingl et al., 2013; Daschner et al., 2018), Australia Bionic Vision's supra-choroidal device (Ayton et al., 2014), Pixium Vision's IRIS II (Hornig et al., 2017), and the more recent subretinal PRIMA (Palanker et al., 2020).

While clinical results indicate patient progress in light detection, object classification, and large letter recognition, the visual resolution of these devices remains limited, making even simple object recognition challenging (Ayton et al., 2020). Essential capabilities such as facial recognition and color judgment are not yet achievable with these devices.

Snellen acuity is a common measure of visual acuity, where 20/20 denotes normal vision, and 20/200 indicates legal blindness. In clinical trials, the highest reported visual acuity for various devices so far is 20/1,260 from Argus II (Humayun et al., 2012), 20/546 from Alpha AMS (Daschner et al., 2018), 20/460 from PRIMA (Palanker et al., 2020), and 20/4,242 from Australian Bionic Vision (Ayton et al., 2014). As shown in Table 1, the visual effects generated by all artificial vision devices are still relatively low. The visual acuity and color discrimination abilities of these devices fall within the legally defined ranges of blindness.

**Table 1:** Comparison of various artificial vision aid devices and techniques.

**Device**	**Concept**	**Number of electrodes**	**Visual acuity**	**Visual field**	**References**
Argus II	Epiretinal	60	20/1,260	19°	Humayun et al., 2012
IRIS II	Epiretinal	150	2.3 logMAR	NA	Hornig et al., 2017
Epiret III	Epiretinal	25	NA	NA	Roessler et al., 2009
Alpha-AMS	Subretinal	1,500	20/546	11°	Daschner et al., 2018
PRIMA	Subretinal	378	20/460	10°	Palanker et al., 2020
Australian Bionic	Suprachoroidal	44	20/4,242	12°	Ayton et al., 2014

There are a total of 1.5 million RGCs in the human retina, with the largest soma having a diameter of about 30 um. Researchers have been studying how to make a smaller electrode. However, there are several technical limitations to using higher-density electrode arrays. For example, the impedance of electrodes increases when their size is reduced. High-impedance electrodes require higher voltage stimulation drivers, which consume more power. Many materials do not have suitable electrochemical properties to elicit neural activity within the safe charge injection limit.

The minimum accuracy is not only limited by the size of the electrode but also by limited temporal precision and the unselective activation of different visual pathways. For example, high-frequency repetitive stimulation to retinal cells can lead to loss of responses. In fact, we can easily make electrodes that are close to the size of retinal cells. However, such electrical stimulation does not allow cells and electrodes to respond one-to-one. Further studies on the spatial resolution, temporal resolution, and the selective activation of retinal cells are needed to make a color visual prosthesis with the appropriate resolution.

Additionally, the visual features produced by most artificial vision devices are black-and-white array blocks (Wu et al., 2023), with very few experiments resulting in color phosphene for subjects (Stanga et al., 2012). Although certain research endeavors have achieved colored phosphene by adjusting the stimulus parameters (Schmidt et al., 1996; Paknahad et al., 2021; Yue et al., 2021), as of now, there is no implantable artificial vision device (globally) capable of generating controlled artificial color vision. Therefore, how to design a controllable artificial color device has been a very important issue for researchers all over the world.

More reviews of artificial visual-assistive technology are available (Hornig et al., 2017; Fernandez, 2018; Wang and Kuriyan, 2020; Lin et al., 2021; Borda and Ghezzi, 2022).

### 3.2 Non-implantable visual-assistive devices

Sensory substitution holds promise for visual rehabilitation among the visually impaired and blind (Maidenbaum et al., 2014). A sensory substitution device (SSD) is designed to convey visual information to the visually impaired by systematically translating visual data into one of their remaining senses. Typically, visual information is transformed into auditory or tactile sensations. For instance, visual data may be translated into voice (Auvray et al., 2007), music (Abboud et al., 2014), vibration (Song et al., 2024), electrical stimulation (Kajimoto et al., 2002; Nau et al., 2012), or a combination thereof. These sensory inputs are intricately coded to convey visual details to the blind individual, including color perception, distance estimation, and object recognition.

Sensory substitution typically relies on simpler sensing devices, such as computer vision or ultrasonic sensors, to acquire visual data. This offers the advantages of affordability, the absence of surgical risk and complications, and greater acceptance among the blind. However, the visual information provided is subject to the limitations of processing algorithms, resulting in reduced visual content and increased difficulty for patients to learn and operate. The device tends to be large in size with limited portability. More seriously, this approach takes possession of the remaining senses of the blind individual and does not necessarily improve vision, thereby heightening the risk in tasks such as navigation and wayfinding. In summary, the advancement of SSDs hinges largely on future advancements in computer vision, device integration, and sensor co-ordination.

Conversely, implantable visual aids typically offer improved vision and object recognition capabilities. Visual perception facilitated by electrical stimulation is easier to acquire later in life. The setup and operation are straightforward. Moreover, the devices are portable and not easy to find. However, due to the necessity of surgical implantation, patients are exposed to surgical risks and complications, and the higher cost leads to reduced patient acceptance. However, supra-choroidal prostheses are anticipated to propel research in the near future due to their minimally invasive nature, making them a viable clinical option. While cortical visual prostheses and some retinal prostheses remain largely experimental and uncertain in terms of the risk-benefit balance, other retinal prostheses appear more promising in terms of advancing the development of a bionic eye.

In fact, color is only a small part of visual information. Furthermore, visual information encompasses shape, contour, distance, and additional elements. Providing solely color information, as in Abboud et al. (2014), is not enough for blind people. Nonetheless, when combined with SSDs and other implantable visual aids, it become a valuable supplementary technology.

### 3.3 Other work toward artificial color vision

Alternative strategies for visual restoration in photoreceptor pathologies involve genetically modifying RGCs to express light-gated ion channels, rendering them directly responsive to light. This application was among the earliest proposed uses of optogenetics (Bi et al., 2006). Recent research indicates that the adeno-associated virus (AAVs)-mediated optogenetic stimulation of RGCs can activate the primate visual cortex (McGregor et al., 2020; Gauvain et al., 2021; Chaffiol et al., 2022). Through this genetically altered cellular approach, RGCs, which lack inherent photosensitivity, can acquire the ability to respond to light. Clinical trials are currently assessing methods for vision restoration, enabling patients to perform object localization to some extent (Sahel et al., 2021). Several other gene therapy strategies have aimed to sensitize RGCs directly to ambient light levels, although they face challenges related to the slow kinetics of these responses (Lin et al., 2008; Berry et al., 2019). An alternative method involves stimulating light-sensitive RGCs by implanting light-emitting arrays, such as micro-LEDs, directly into the eye, potentially enabling the attainment of artificial color vision.

In neuroscience and medical applications, it is essential to integrate sensory regions with high-density sensors (i.e., a large sensor count) (Stringer et al., 2019; Liu et al., 2022). Emerging materials such as nanomaterials and organic semiconductors present opportunities for this integration. Recent advancements in materials and nano-engineering have enabled the exploration of novel approaches to neuronal interactions beyond traditional metal-based electrode stimulation methods. Materials such as carbon nanotubes (David-Pur et al., 2014), graphene (Yang et al., 2021), nanocrystalline diamond (Hadjinicolaou et al., 2012), and silicon nanowires (Ha et al., 2016) enhance the electrochemical properties and mechanical connectivity of neuro-electrodes through distinctive surface shapes and charge injection techniques. Integrating these materials into retinal prostheses or VCPs could improve color discrimination and fidelity.

Organic semiconductors hold promise for neural interfaces owing to their biocompatibility, soft mechanical properties, and mixed electron/ion conduction. Planar poly[3-hexylthiophene] (P3HT) (Maya-Vetencourt et al., 2017) devices and subretinally injected P3HT nanoparticle formulations (Jakešová et al., 2019) have partially restored vision *in vivo* in rodent models of photoreceptor degeneration. Theoretical *ex vivo* models demonstrate the restoration of visual acuity to 20/480 with retinal coverage equivalent to a 43° viewing angle (Chenais et al., 2021). An additional fascinating prospect for retinal prostheses using organic semiconductors involves exploiting the narrow band-gap of photoactive material to react to particular wavelengths within the visible spectrum, thereby emulating the light response of cone cells in the retina (Sherwood et al., 2023). By integrating organic semiconductor materials with absorption spectra across short, medium, and long wavelengths, electrical stimulation in the retina may respond to light of varying frequencies, enabling artificial color vision.

The advancement in perovskite materials has offered fresh perspectives on subretinal light-sensing stimulus arrays. Hou et al. devised a narrow-band light-sensitive sensing array, incorporating red, green, and blue colors by employing perovskite (Hou et al., 2023). In contrast to prior sensors utilized in subretinal implants, this narrow-band imaging sensor eliminates the need for complex optical filter arrays and operates without external biasing, facilitating power-free light-detection capabilities. Long et al. (2023) proposed a similar concept to develop a neuromorphic bionic eye capable of color vision without filters, employing perovskite nanowires. It presents a novel avenue for enhancing the integration of color vision prostheses in subretinal implants.

## 4 Discussion

### 4.1 The effect of the presence of rods on color discrimination

In the human visual system, attention is often focused on the central field of view, particularly the macular area, which is crucial for detailed object recognition, color discrimination, and text recognition.

However, the peripheral field of view is also critically important. This larger part of our visual field is used for sensing surroundings and monitoring movement. The distribution of photoreceptors reveals that the cone density in the central field of view, especially near the macular area, is very high, reaching up to 15,000 per mm^2^ (Curcio et al., 1990). As the visual angle increases, the number of cones sharply decreases while the number of rods increases, surpassing the maximum density of cones.

In terms of color discrimination, cones play a leading role. Recent studies have raised a puzzling issue: although color discrimination diminishes with an increased peripheral angle of view, the decline is not as steep as the reduction in cone density (Tyler, 2015, 2016; Lin et al., 2023; Rozhkova et al., 2023).

**Figure 8:**
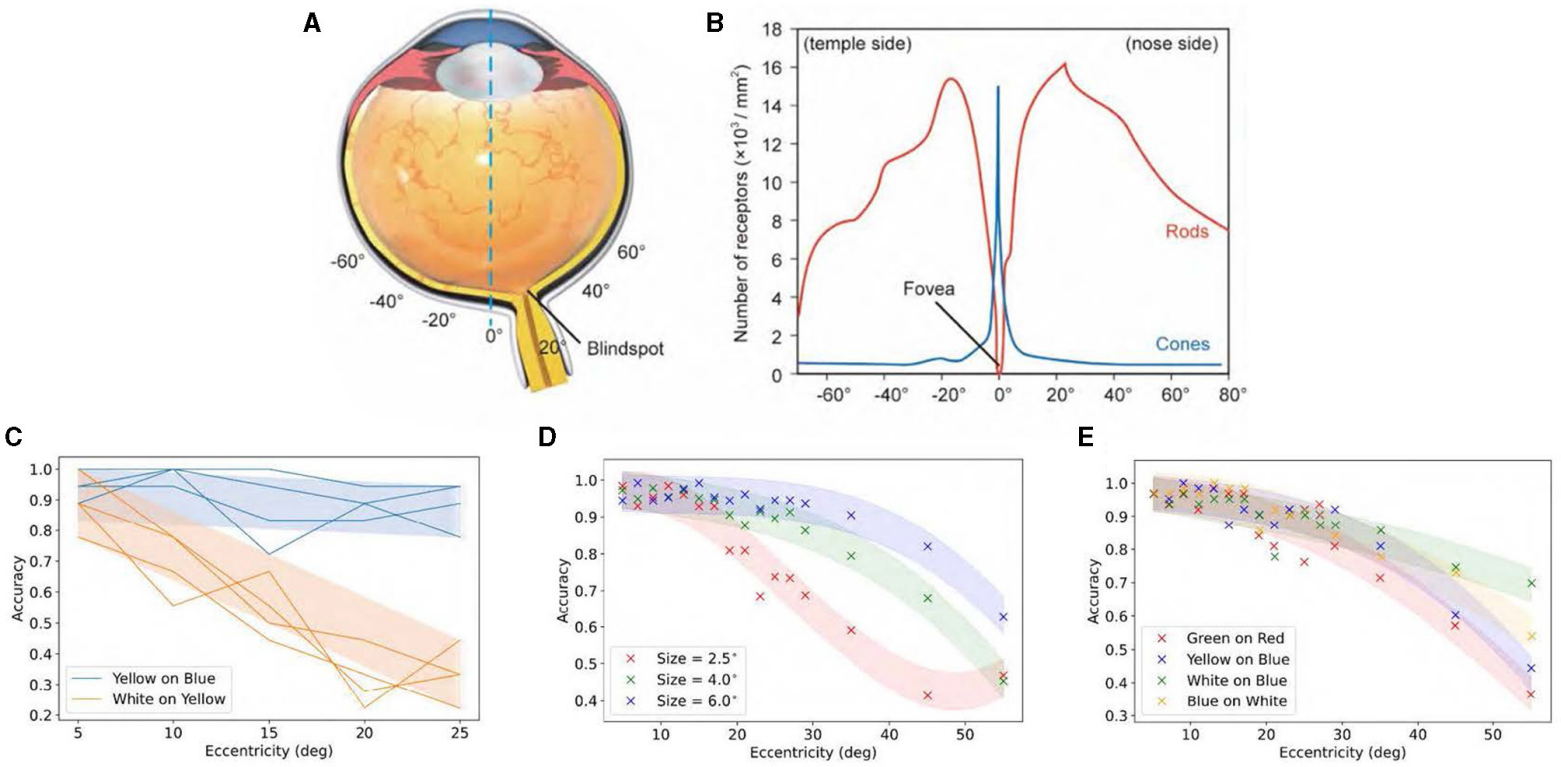
Relationship between color discrimination ability and eccentricity angle. **(A)** Schematic structure of an eyeball. **(B)** Density distributions of cones and rods along the retina (blind spot ignored). **(C)** Recognition accuracy of yellow words on blue background and blue words on yellow background with eccentricity angle. **(D)** Recognition accuracy of Chinese characters with various colors is assessed against different backgrounds and horizontal eccentricities. **(E)** Recognition accuracy of digits with various colors is assessed against different backgrounds and horizontal eccentricities. Adapted from Lin et al. (2023).

Figure 8 illustrates that the density of cone cells decreases from 15, 000/*mm*^2^ at the fovea to <1, 000/*mm*^2^ at 20° away from the central axis. However, when comparing it to color discrimination accuracy at 20°, no proportional decrease was observed in cone cell density. Surprisingly, color discrimination in the peripheral vision remains robust, contradicting the belief that color is determined by cones.

This fact is a contradiction to the model that color vision is mainly determined by cones. It may stem from the visual system's complex complementary mechanisms involving multiple cell types and brain regions in color discrimination. Rods are particularly important in this regard. Even though cone numbers decrease in the peripheral vision, the relative abundance of rods may help bridge this gap. Notably, some color perception persists, even in conditions where only rods are active. These perceptions seem to arise from higher cognitive activities that infer color from black-and-white inputs, drawing on prior experiences in nature (Pokorny et al., 2006; Zele and Cao, 2015).

An interesting aspect is that the visual system's complexity goes beyond the function of any single cell type. Color perception likely results from the combined actions of various cell types and brain regions. Processing in the visual cortex and interactions among different visual channels might bolster color discrimination in peripheral vision.

In mammals, evidence suggests that rods and cones inhibit each other. In order to adapt to low-light conditions, rods inhibit the activity of cones (Rodieck and Rushton, 1976; Eysteinsson and Frumkes, 1989). Studies on the mudpuppy suggest that this inhibition originates from horizontal cells, which receive input from rods and, subsequently, inhibit cones (Eysteinsson and Frumkes, 1989). In humans, the monochromatic vision associated with S cones still enables color vision in low light, suggesting possible opponency between rods and S cones.

The interaction between cones and rods has been directly demonstrated by specific kinds of RGCs in the mouse retina (Reitner et al., 1991). However, there seems to be no cell type in primates homologous to this mouse ganglion cell. To date, there is no evidence to suggest that rods are opponent to S cones or that rods are opponent to M or L cones in primate RGCs. Psychophysically, experimental evidence has shown a non-linear combination of signals from rods and cones. This may result in a decrease in yellow perception or an increase in blue perception. However, this finding is inconsistent with the linear relationship in current opponent-color models. The discovery that rod and S cone cells input to small, bi-stratified S ON cells in the primate retina (Crook et al., 2009; Field et al., 2009) suggests that rods might enhance cone-based color discrimination (Buck, 1985, 2014). In conclusion, although mammalian rod circuits are well-documented, the specific retinal circuits of rod cells that affect human color vision still require further clarification.

### 4.2 The encoding of color information by RGCs

The human visual system's remarkable ability to discriminate between colors is an astounding phenomenon. Indeed, the human visual system is known for its high color resolution at about 10 million different colors (Judd et al., 1963). This capability raises questions about how such high-resolution color discrimination is achieved. At least three different types of photoreceptors are necessary to meet the minimum requirements for distinguishing colors (Conway, 2009).

In essence, for the smallest pixel (a single cell) of the human eye, at least three different bands of intensity information are typically required to distinguish the color wavelength. However, for bipolar and RGCs following the photoreceptors, color information transmission does not occur via three separate channels for each color wavelength but rather through a mechanism of color opponency.

Current anatomical and physiological understanding shows that color discrimination relies not only on the type of photoreceptor cells but also on the complex processes of information transmission and processing. Bipolar and horizontal cells perform multi-level integration and processing on the signals from cone and rod cells. These signals from various photoreceptors intertwine before converging in RGCs. If an RGC serves as the smallest unit for transmitting pixel color, then color information should not be transmitted through three separate channels for different wavelengths, but rather, it should be integrated into a single channel. This single-channel encodes the information from these different wavelengths to transmit the color signal to the brain efficiently and accurately.

In neuroscience, RGCs have been notably successful as feature detectors within the visual system. Studies show that these cells demonstrate specific sensitivity to various visual features, including motion, shape, and color (Gollisch and Meister, 2010; Sanes and Masland, 2015; Baden et al., 2016). This specificity is primarily determined by the que spatial, spectral, and temporal properties of the receptive fields of RGCs, rendering them highly selective to different visual information. In RGCs, different colors exhibit distinct spatiotemporal coding patterns. Like other nerve cells, RGCs encode information in a binary manner, transmitting it to the brain as all-or-nothing action potentials. However, if the brain only relies on the response of a single cell for chromaticity recognition, achieving accurate judgment becomes difficult. Therefore, this signal coding should reflect not only waveform differences but also subtle variations in amplitude, frequency, duration, and time series.

### 4.3 Color vision perception by electrical stimulation encoding

In contemporary clinical devices, electrical current diffuses from the stimulating electrode to the distant return electrode, leading to the simultaneous activation of multiple cells in the distribution area (Tong et al., 2020). All electrodes stimulate neurons similarly, activating various ganglion cells simultaneously, which is a process quite different from the image processing of a healthy retina. The irregular and poorly controlled phosphenes, resulting from non-selective and broad electrical stimulation, significantly limit the imaging quality of artificial vision. Most patients describe this sensation as resembling a candle or light bulb, typically perceiving white, blue, or yellow (Hornig et al., 2017). In fewer instances, colors such as red, green, or black are reported. Typically, subjects reported only a single color. Only a few patients experienced phosphenes in multiple colors.

Recently, researchers have discovered that adjusting electrical parameters may hold the potential for restoring color vision in the blind (Yue et al., 2021). Stanga et al. (2011) discovered that altering electrical stimulation parameters could induce color vision in patients. In clinical trials involving nine patients with retinitis pigmentosa, the Argus II device enabled them to perceive eight different colors: orange, yellow, blue, green, pink, gold, purple, and brown. Additionally, this color perception was repeatable. The following year, tests on four patients with retinitis pigmentosa using Argus II showed it was possible to induce the simultaneous perception of two different colors. These patients perceived seven different combinations of color: gray-white, yellow-gray, orange-white, white-blue, brown-white, yellow-white, and yellow-blue (Stanga et al., 2012).

A recent study tested adjusting the frequency of electrical stimulation to alter color perception in blind individuals (Yue et al., 2021). Five out of seven subjects experienced a shift toward blue-purple when the frequency increased from 6 to 120 Hz. Similar findings were made by Schmidt et al. (1996), who proposed that stimulus intensity influences color changes, with higher intensity tending toward white (Towle et al., 2021). Subsequently, Paknahad et al. designed an experiment proposing an “amplitude-frequency” stimulation strategy. This strategy uses computational tools to predict the color coding of retinal visual prostheses and has been validated by experimental data (Paknahad et al., 2021).

Although fMRI has been employed to study the color-sensitive region in the human brain's posterior section, the detailed structure of this region in humans and primates, its spatial distribution, and the cortical color response to retinal prostheses remain poorly understood (Lu and Roe, 2008). The study of color-visual prostheses has a long way to go. Fortunately, color perception does not need to precisely replicate a camera-defined object's actual color. The effective use of external devices can still offer an additional dimension of visual perception to the blind.

A recent report showed that users of a retinal vision prosthesis could better distinguish crowded pedestrians using an infrared camera compared to a conventional camera (Sadeghi et al., 2021). Special coding allows patients to be informed about the corresponding light stimulus of different color frequencies, essentially the “color attribute” assigned to objects by artificial vision devices. This approach focuses on technological innovation and aligns with biological principles, aiming to provide a more intelligent visual experience.

The introduction of coding strategies into artificial vision is undeniably set to enhance their functionality and utility significantly. This approach not only aids individuals with visual impairments in perceiving and understanding the world in color but also holds immense potential for advancing future medical research and technological innovation. With a deeper understanding of color neural mechanisms and ongoing advances in medical technology, we anticipate further remarkable breakthroughs in artificial color vision in the near future.

### 4.4 Interventional strategies for artificial color vision treatment

In order to effectively implement artificial color vision in patients with different conditions of blindness, it is necessary to tailor treatment strategies to specific neural surrogates and cognitive characteristics.

Brain plasticity is influenced by the age of onset of blindness in individuals (Bedny et al., 2012; Bang et al., 2023). A potential explanation lies in cholinergic signaling originating from the basal nucleus of Meynert, which contributes to varying levels of plasticity. Remarkably, the nucleus basalis of Meynert exhibits distinct functional connectivity patterns in individuals with early vs. late-onset blindness. This enhanced connectivity occurs at both global and local levels, including visual, language, and default mode networks. Late-life vision loss generally leads to reduced plasticity compared to congenital blindness, due to age-related declines in brain reorganization capacity. Therefore, interventions aimed at restoring artificial color vision may be more effective in younger individuals, allowing for better adaptation and functional reorganization of the cerebral cortex.

In congenital blindness, tactile and auditory information can be reinterpreted by the brain to compensate for visual information through mechanisms of brain plasticity triggered by training (Chebat et al., 2020). Colored artificial vision systems should first focus on augmenting tactile and auditory sensory substitution devices to enhance brain plasticity and cortical processing of spatial information before gradually introducing color cues. This approach utilizes enhanced plasticity and cholinergic influences to allow the brain to effectively adapt to new visual inputs.

Early visual experience shows great importance in color processing development (Vogelsang et al., 2024). This study highlights the importance of early visual experience and developmental trajectories in building robust visual recognition systems. Simulations of neural responses based on deep convolutional neural networks also confirmed that by incremental training from grayscale to color images, they outperformed deep convolutional neural networks trained with color images only. For artificial color vision, this suggests that mimicking human developmental stages—starting with limited color inputs and gradually introducing full-color data—could improve the adaptability and generalization of artificial vision systems. Especially for people with early blindness, introducing color images after initial grayscale training may help the brain integrate color information.

### 4.5 Detection of color vision

In order to clarify color vision perception, employing a multifaceted approach that includes electro-retinography (ERG), fMRI, and visual evoked potentials (VEPs) provides a comprehensive perspective. ERG captures the retina's electrical response to light stimulation, typically manifesting as a biphasic negative/positive waveform when there is a flash of light or a pattern appears brightly (Creel, 2019). This response is recorded using an active extracellular electrode, which can be placed on the cornea, in the vitreous, or at various depths within the retina. Through the analysis of these electro-physiological waveforms, it becomes possible to determine the response levels of different retinal cells. Consequently, the retina's perception of color information can be effectively analyzed by examining the response level of photoreceptors.

However, ERG measurement is only suitable for optical stimulation and not for artificial visual prostheses that directly stimulate RGCs electrically. Should implantable optical vision prostheses become available, ERG might serve as an effective means to assess color perception. fMRI serves as a potent tool for investigating the neural correlates of color perception. By detecting changes in brain blood oxygen levels, it can effectively map and quantify brain responses associated with color perception (Morita et al., 2004). However, fMRI is limited to analyzing patterns of activity in various brain regions and cannot directly assess the frequency of color perceived by the brain. VEPs play a crucial role in diagnosing visual nerve diseases (Odom et al., 2004). These potentials enable the diagnosis and detection of specific diseases by measuring and evaluating the brain's response to specific stimuli. In comparison to fMRI, VEPs offer advantages such as high temporal resolution, repeatability, and high signal quality. Studies conducted by Tello et al. (2015); Chu et al. (2017); Duart et al. (2021) delved into the brain responses elicited by different colors, revealing variations in the latency and shape of VEPs induced by distinct color frequencies. Building upon their foundation, (Böck et al., 2021) employed an artificial neural network to analyze VEPs for the online monitoring of an individual's color discrimination ability. This endeavor provided evidence supporting the feasibility of utilizing VEPs for the automatic classification of color perception. In other words, this technology has the potential to predict the colors perceived by animals in the future. Such technology serves as a valuable tool for verifying the visual effects generated by artificial vision devices in clinical practice.

## 5 Some new concepts and ideas

We have reviewed the comprehensive understanding of the physiological structure and functioning of the human retina and the noteworthy breakthroughs in the field of artificial vision. While the development of these technologies brings hope to individuals with visual impairments, significant challenges and opportunities for improvement persist.

Building on the understanding of retina and eye physiology, we propose innovative visions for artificial vision devices. These concepts aim to propel the advancement of artificial vision devices, ultimately enhancing the quality of life for individuals with visual impairment. By amalgamating existing medical and engineering knowledge, we anticipate paving the way for future research and innovation, further improving and broadening the areas of application of artificial vision technologies.

### 5.1 Artificial vision system imagination system based on electronic encoding stimulus array

This system utilizes a 3D electrode array composed of a biologically compatible flexible substrate, specifically designed for implantation in a wide area around the subretinal macular region, covering an area of 10–30 *mm*^2^. The device comprises a transparent substrate and 1,000–10,000 micro-electrodes arranged in a hexagonal or square pattern on the substrate. The pixel spacing is 10–30 μ*m*, and the height of the conical electrode ranges from 10–50 μ*m* to match the size of a single or a small number of ganglion cells. Post-implantation, these conical electrodes establish close contact with RGCs.

The uniqueness of this system lies in its capability to transmit electrical stimulation signals with different waveforms, frequencies, delay times, and amplitudes to retinal ganglion cells, thereby activating them to generate various encoded responses. According to the contents of Sections 2.2 and 3.3, it has been demonstrated that ganglion cells employ different encoding strategies for various visual stimuli (Adrian and Zotterman, 1926; Ishikane et al., 2005; Thiel et al., 2006; Risner et al., 2010). Different visual features lead RGCs to produce different spatiotemporal responses. There is also evidence that Electrical stimulation with spatial and temporal variations can induce phosphenes with color in patients (Towle et al., 2021; Yue et al., 2021). It is therefore possible to use different spatial and temporal codes to stimulate specific retinal cells to produce colored visual features. Moreover, machine learning can supervise VEPs, enabling adaptive adjustment of stimulation parameters based on VEP waveforms. This approach facilitates the generation of controlled artificial color vision for the blind.

Currently, aerosol jet printing technology has reached a relatively advanced stage, enabling the commercial mass production of conical electrode ranging from several microns to tens of microns in size (Secor, 2018). This advancement raises the prospect of developing a low-cost artificial vision device capable of providing color vision to the blind in the future. As this field continues to progress, this technology is expected to play a crucial role in enhancing the quality of life for the blind.

### 5.2 “Eye in Eye” based on reflection

**Figure 9:**
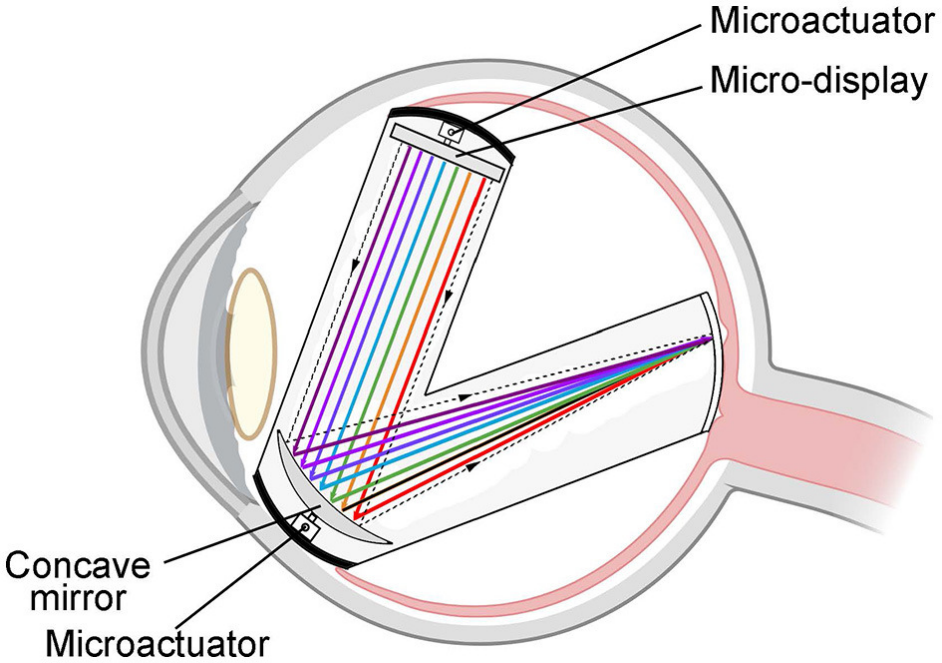
Schematic diagram of the “eye in eye” based on reflection. The device consists of a concave reflector and a micro-display encapsulated in a container. By utilizing the integrated micro-display and concave mirror, the device precisely projects colored light into the fovea of the retina.

This device is a specifically designed color vision restoration device implanted inside the vitreous body of the eyeball. By utilizing an integrated micro-display and concave mirror, the device precisely projects colored light into the fovea of the retina. This process facilitates the restoration of color vision in the visually impaired. The device employs a micro-actuator to regulate the position and angle of both the miniature display and concave mirror, allowing for the flexible adjustment of image position and sharpness within the retinal region, as shown in Figure 9. With a diagonal measurement of 0.13 inches and a total pixel count of more than 200,000, the micro-display allows for the one-to-one projection of each pixel point onto the retinal photoreceptors.

The micro-display can present real-time images of the surrounding world captured by external cameras or images from visual enhancement devices such as infrared and ultraviolet cameras. Additionally, the device supports displaying various types of information, such as text, images, or videos transmitted through the network cloud. Its flexibility and versatility allow patients to customize settings based on their specific needs, facilitating better adaptation to different environments and tasks. The micro-display employs the established micro-LED processing method, and its reflective mirror surface is produced through a mature coating process, which imparts it with the benefits of a simple structure and mature technology. Consequently, it presents a novel and effective approach to visual restoration and enhancement, offering a more convenient and practical solution for the visually impaired.

### 5.3 A flexible micro-display device implanted in front of the retina

**Figure 10:**
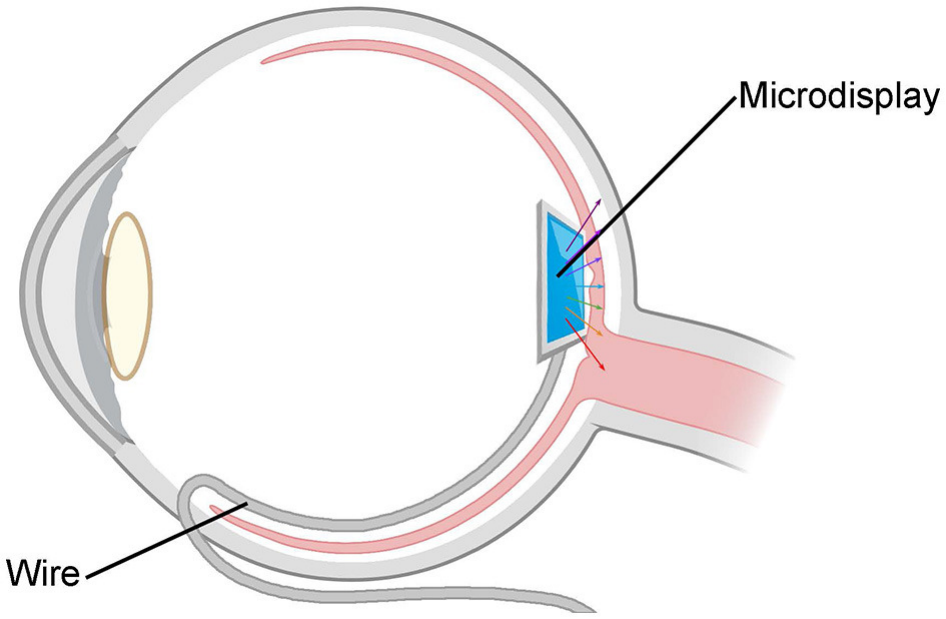
Schematic diagram of the flexible micro-display device. The flexible micro-display is positioned in front of the retina. It utilizes an external image-receiving module capable of receiving video information from the Internet or local cameras. After compression and encoding via the image-processing module, the processed image is transmitted to the intraocular implantable micro-display screen.

By being positioned in front of the retina, this flexible micro-display device functions as a visual restoration tool (as shown in Figure 10), aiming to provide patients with a colorful visual experience. It is designed to fully utilize the external image-receiving module capable of receiving video information from the Internet or local cameras. The information is then compressed and encoded via the image-processing module. The processed image is transmitted to the intra-ocular implantable micro-display screen, emitting light through micro-LEDs and presenting patients with a color visual scene.

Thanks to the high-density advantage of micro-LEDs, each pixel can be precisely directed to individual retinal photoreceptors in a one-to-one manner. The display area is not limited to a square shape; it can also take on circular and hemispherical forms. The hemispherical screen, mirroring the curvature of the eyeball, completely covers the retina, thereby enabling patients to achieve comprehensive color vision restoration. Furthermore, the display can be configured in a circular ring shape to address the needs of individuals with macular lesions and special requirements. This design facilitates the delivery of visual image information around the retina without affecting the fovea's vision. This device is indicated for patients with partial retinal degeneration in order to project image information onto healthy areas of the retina. More importantly, gene therapy can express light-gated ion channels by modifying RGCs to make them directly sensitive to light (Bi et al., 2006; McGregor et al., 2020; Gauvain et al., 2021; Chaffiol et al., 2022). When coupled with related gene therapies, optical stimulation can be performed directly at cellular resolution to avoid the use of complex extraocular spatial light modulator devices. In the future, it may also be applied to combat populations to enhance their vision. Such innovative adaptability caters to the patient's needs, enhancing user comfort. This concept offers a novel approach for the blind to attain color vision and lays the groundwork for vision enhancement.

An important consideration regarding implant safety is the mechanical design of intraocular devices. Devices have the potential to damage tissue mechanically, chemically, and thermally (Opie et al., 2012). Therefore, materials that come into long-term contact with such fragile biological tissue must also have a Young's modulus to avoid tearing, crushing, or separating the retina. Typically polymers like polydimethylsiloxane (PDMS) (Lötters et al., 1997), polymethyl methacrylate (PMMA) (Suaning et al., 2014), polyimide (PI) (Ghosh, 1996) and cyclic olefin copolymer (COC) (Baek et al., 2020) and other such polymers are good common encapsulation materials for implantable devices. In this device, PDMS, colorless transparent polyimide (CPI) (Baek and Seo, 2022) and COC are superior to other materials in terms of optical transparency. Considering the excellent encapsulation properties of COC due to its thermoplasticity and its ability to be fabricated into microlens arrays (Baek et al., 2021), we believe that COC is worthy of further in-depth study to further advance the field of optical retinal prostheses.

## 6 Conclusion

In short, researchers believed that creating color vision was a complicated process that entailed a series of hierarchical steps. Experiments showed that the retina may involve three types of cones, each sensitive to different light wavelengths. Outputs from RGCs may undergo initial processes in the LGN before reaching the V1. The double-opponent cells in V1 may strongly respond to color contrast, and color-sensitive cells in V1 are mainly located in CO patches. Cells in the CO patch of V1 project to those in V2, where they exhibit enhanced selectivity for the hue of color vision. Color constancy, the stable perception of object color under varying lighting, may emerge in advanced visual cortex areas, e.g., V4.

However, many questions about color vision remain open. An in-depth exploration of color vision processing may offer a theoretical framework for developing novel visual prostheses. For instance, some direct, personal experiences from a group of patients who had been implanted with visual prostheses that worked using electrode arrays releasing electrical pulses showed that a combination of external electrical pulses that are varied in time sequence and field intensity could also result in blurred color vision.

This suggested that the coding of the neural signals in the optical nerve, which transmit from the retina to various levels of the visual cortex, may also play a key role in human color vision. These changing codes for different colors may originate from the same group of cone or rod cells but are regulated by varying incident color lights. Thus, by optimizing the encoding patterns of the electrical stimulus of a visual prosthesis, it is highly possible to achieve ideal artificial color vision.

We also propose the utilization of minimized optical devices implanted in the eyes to project clear color images directly onto the targeted regions of the retina. This approach may open up a new avenue for restoring color vision in individuals with visual impairments, such as macular disease.

## Author contributions

BZ: Conceptualization, Data curation, Investigation, Methodology, Visualization, Writing – original draft, Writing – review & editing. RZ: Data curation, Visualization, Writing – original draft. JZ: Data curation, Investigation, Methodology, Visualization, Writing – original draft. JY: Conceptualization, Investigation, Methodology, Supervision, Validation, Writing – review & editing. SX: Conceptualization, Funding acquisition, Supervision, Validation, Writing – review & editing.

## Footnotes

^1^ https://worldblindunion.org/
